# Draft Genome Sequence of Blood Disease Bacterium A2 HR-MARDI, a Pathogen Causing Banana Bacterial Wilt

**DOI:** 10.1128/genomeA.00408-17

**Published:** 2017-06-01

**Authors:** Rafidah Badrun, Norliza Abu Bakar, Rozeita Laboh, Rohaiza Redzuan, Indu Bala Jaganath

**Affiliations:** a Biotechnology & Nanotechnology Research Centre, Malaysian Agricultural Research and Development Institute (MARDI), Serdang, Malaysia; b Crop & Soil Science Research Centre, Malaysian Agricultural Research and Development Institute (MARDI), Serdang, Malaysia

## Abstract

Blood disease bacterium A2 HR-MARDI was isolated from banana plants infected with banana blood disease and which were planted in Kuala Kangsar, Malaysia. Here, we report a draft genome sequence of blood disease bacterium A2 HR-MARDI, which could provide important information on the virulence mechanism of this pathogen.

## GENOME ANNOUNCEMENT

Blood disease bacterium (BDB) is a Gram-negative rod and slow-growing bacterium. It belongs to phylotype IV of the *Ralstonia solanacaerum* species complex (1). It is closely related to but distinct from the bacterium that causes Moko disease, although the symptoms produced by their infections are slightly similar (2). Unlike Moko disease pathogen, BDB is not pathogenic to *Heliconia* spp. in the wild and to solanaceous hosts following artificial inoculation (3). BDB was later considered to be an aberrant form of Moko (4). The name blood disease was originally adopted because of the appearance of thick milky white droplets, yellow, or red-brown liquid that oozes out of the vascular tissues of the infected plant at cut and wounded plant surfaces. Blood disease is estimated to be spreading at the rate of approximately 25 km per year (5).

Blood disease bacterium A2 HR-MARDI was identified using a bioassay and a pathogenicity test. The identity of this bacterium was also confirmed using 16S rRNA gene cloning. The genome was sequenced using PacBio RS II sequencing platform, which generated 2 single-molecule real-time (SMRT) cells of sequencing data of 20 kb. The genome assembly was carried out by using PacBio Hierarchical Genome Assembly Process 2.0 (HGAP 2.0). The final assembly of the 2 contigs made up single 3,603,619-bp chromosome and single 1,486,041-bp megaplasmid sequences. The total BDB genome length is 5,089,660 bp, with a mean G+C content of 66.4%. Gene models of BDB A2 HR-MARDI were predicted by using *ab initio* software Prodigal version 2.60. There were 3,276 predicted coding sequences (CDSs) (≥30 amino acids [aa]) found in the chromosome, whereas 1,340 CDSs were found in the megaplasmid. A total of 54 copies of tRNA and 9 copies of rRNA were predicted by using tRNAscan-SE 1.3.1 and rRNAmmer-1.2, respectively.

The identification of potential virulence factors was carried out by blast analysis against the Pathogen-Host Interactions database (PHI-base). A total of 17 genes were identified as potential virulence factors, with 13 genes found in the chromosome and 4 genes in the megaplasmid. Further analysis of gene annotations was also performed with Rapid Annotations using Subsystems Technology (RAST) (6). From the RAST results, the draft genome contains 4,654 coding sequences, 436 subsystems, and 63 RNAs. The extensive data mining from genome analysis has revealed a list of effector/*hrp* genes. The putative effector genes found in the genome are *hrpB, hrpW, popB, popC, hrpA, hrpH, popA1, hrpJ, hrpX, hrpY prhJ*, and *popW*. The draft genome sequence of BDB A2 HR-MARDI will provide insights into the discovery of more potential virulence factor and hypersensitive response and pathogenicity (*hrp*) genes from the pathogen.

### Accession number(s).

The genome sequence of blood disease bacterium A2 HR-MARDI has been deposited in GenBank under the accession numbers CP019911 (chromosome) and CP019912 (plasmid).
